# Prevalence of Infection-Competent Serogroup 6 *Legionella pneumophila* within Premise Plumbing in Southeast Michigan

**DOI:** 10.1128/mBio.00016-18

**Published:** 2018-02-06

**Authors:** Brenda G. Byrne, Sarah McColm, Shawn P. McElmurry, Paul E. Kilgore, Joanne Sobeck, Rick Sadler, Nancy G. Love, Michele S. Swanson

**Affiliations:** a Department of Microbiology and Immunology, University of Michigan, Ann Arbor, Michigan, USA; b Department of Civil and Environmental Engineering, Wayne State University, Detroit, Michigan, USA; c Department of Pharmacy Practice, Wayne State University, Detroit, Michigan, USA; d School of Social Work, Wayne State University, Detroit, Michigan, USA; e Department of Family Medicine, Michigan State University, Flint, Michigan, USA; f Department of Civil and Environmental Engineering, University of Michigan, Ann Arbor, Michigan, USA; University of Chicago

**Keywords:** Flint water crisis, Legionnaires' disease, intracellular pathogen, macrophages, outbreak

## Abstract

Coinciding with major changes to its municipal water system, Flint, MI, endured Legionnaires’ disease outbreaks in 2014 and 2015. By sampling premise plumbing in Flint in the fall of 2016, we found that 12% of homes harbored legionellae, a frequency similar to that in residences in neighboring areas. To evaluate the genetic diversity of *Legionella pneumophila* in Southeast Michigan, we determined the sequence type (ST) and serogroup (SG) of the 18 residential isolates from Flint and Detroit, MI, and the 33 clinical isolates submitted by hospitals in three area counties in 2013 to 2016. Common to one environmental and four clinical samples were strains of *L. pneumophila* SG1 and ST1, the most prevalent ST worldwide. Among the Flint premise plumbing isolates, 14 of 16 strains were of ST367 and ST461, two closely related SG6 strain types isolated previously from patients and corresponding environmental samples. Each of the representative SG1 clinical strains and SG6 environmental isolates from Southeast Michigan infected and survived within macrophage cultures at least as well as a virulent laboratory strain, as judged by microscopy and by enumerating CFU. Likewise, 72 h after infection, the yield of viable-cell counts increased >100-fold for each of the representative SG1 clinical isolates, Flint premise plumbing SG6 ST367 and -461 isolates, and two Detroit residential isolates. We verified by immunostaining that SG1-specific antibody does not cross-react with the SG6 *L. pneumophila* environmental strains. Because the widely used urinary antigen diagnostic test does not readily detect non-SG1 *L. pneumophila*, Legionnaires’ disease caused by SG6 *L. pneumophila* is likely underreported worldwide.

## IMPORTANCE

*L. pneumophila* is the leading cause of disease outbreaks associated with drinking water in the United States. Compared to what is known of the established risks of colonization within hospitals and hotels, relatively little is known about residential exposure to *L. pneumophila*. One year after two outbreaks of Legionnaires’ disease in Genesee County, MI, that coincided with damage to the Flint municipal water system, our multidisciplinary team launched an environmental surveillance and laboratory research campaign aimed at informing risk management strategies to provide safe public water supplies. The most prevalent *L. pneumophila* strains isolated from residential plumbing were closely related strains of SG6. In laboratory tests of virulence, the SG6 environmental isolates resembled SG1 clinical strains, yet they are not readily detected by the common diagnostic urinary antigen test, which is specific for SG1. Therefore, our study complements the existing epidemiological literature indicating that Legionnaires’ disease due to non-SG1 strains is underreported around the globe.

## INTRODUCTION

The leading cause of disease due to drinking water in the United States is *Legionella pneumophila* (1). This bacterium naturally resides in fresh water, but the majority of Legionnaires’ disease cases originate in engineered water systems. People become infected with legionellae by inhaling contaminated aerosols generated by devices that release water vapors, such as cooling towers, hot tubs, whirlpools, decorative fountains, and showers (2). Older adults with underlying disease, immunosuppression, or a history of smoking are especially vulnerable to Legionnaires’ disease, a severe, sometimes fatal, pneumonia (3). For reasons not understood, the incidence of Legionnaires’ disease increased 3-fold from 2000 to 2011 across the United States and all age groups (1, 3, 4). Likewise, the cases reported in Europe tripled during the 1995–2014 period (5).

Clinicians typically treat community-acquired pneumonia empirically and promptly with broad-spectrum antibiotics to forgo the expense and time required for specific diagnosis (3, 6). When the infectious agent is sought, the most common diagnostic tool for Legionnaires’ disease is the urinary antigen test, a rapid low-cost assay specific for *L. pneumophila* of serogroup 1 (SG1) (7). Although this pathogenic type of legionellae is associated with >80% of Legionnaires’ disease cases worldwide, >60 species and 16 serogroups exist, half of which have been isolated from patients (8). Disease due to non-SG1 *L. pneumophila* is likely underreported, since in the United States and Europe >75% of Legionnaires’ disease cases are diagnosed by the urinary antigen test and just ~5% are confirmed by culture (8, 9). Indeed, it is estimated that only ~5% of Legionnaires’ disease cases in the United States are reported (10). Although widespread reliance on the urinary antigen test and empirical broad-spectrum antibiotic treatment are efficient and cost-effective for most patients, these clinical practices hamper not only identification of other pathogenic legionellae but also epidemiological investigations to track and eliminate the source of Legionnaires’ disease outbreaks.

Especially prone to colonization by *L. pneumophila* are hospitals and hotels, due to their warm temperatures, large size, and multiple partitions with irregular usage that create areas with low or no flow (11). Stagnant water favors growth of biofilms, adherent microbial communities that are difficult to eradicate (3, 12). *L. pneumophila* also survives and replicates within predatory free-living protozoa that graze on biofilms (11). Extermination of legionellae residing within protozoa or biofilms requires elevated doses of disinfectants (13–17). Consequently, despite remediation efforts, *L. pneumophila* can persist in complex engineered water systems and cause recurrent disease outbreaks for decades (18–23). Indeed, despite infection control measures, *L. pneumophila* was found to colonize 70% of Pittsburgh and 60% of Paris hospital water systems surveyed (24, 25).

Compared to our knowledge of the established risks of legionella colonization within large buildings, relatively little is known about exposure within single-family homes and low-rise housing units. Based on limited sample sizes, the prevalence of residences whose water is culture positive for legionellae has been reported to be 6 to 30% (26–31). *L. pneumophila* colonization can be suppressed by maintaining a chlorine concentration of >2 mg/liter (32, 33). However, hot water residential lines often contain little measurable levels of chlorine, due to its decay at elevated temperatures (32). Since only 4% of the Legionnaires’ disease cases reported in the United States from 2000 to 2009 were associated with outbreaks (4), sporadic or epidemiologically unrelated cases of Legionnaires’ disease due to colonization of the built environment by legionellae may be more insidious and widespread than currently documented.

In the summers of 2014 and 2015, Genesee County, MI, endured outbreaks of Legionnaires’ disease (26). Compounding the anxiety within the community, the 87 confirmed cases of disease occurred during a period of sustained damage to the municipal water system of Flint, the county’s largest city (27). When the anticorrosive agent orthophosphate was omitted from the Flint water supply, toxic lead leached from pipes and fixtures into the municipal water. The impact of the water’s altered physiochemistry on the burden, resilience, or virulence of the microbial communities in the Flint water system is an open question.

To inform the rational design of risk management strategies to keep public water supplies safe, the State of Michigan supported the Flint Area Community Health and Environment Partnership (FACHEP) research team’s analysis of legionellae within the Flint municipal system. Working closely with local health care providers, community organizations, and government agencies, we applied a multidisciplinary strategy that integrated environmental monitoring and water testing with clinical education, molecular epidemiology, and laboratory assays of *L. pneumophila* virulence. Here we present our laboratory analysis of the genetic diversity and virulence potential of *L. pneumophila* isolated from Flint premise plumbing in the fall of 2016.

## RESULTS

### *Legionella* screening in Flint, MI, and neighboring census tracts.

**FIG 1:**
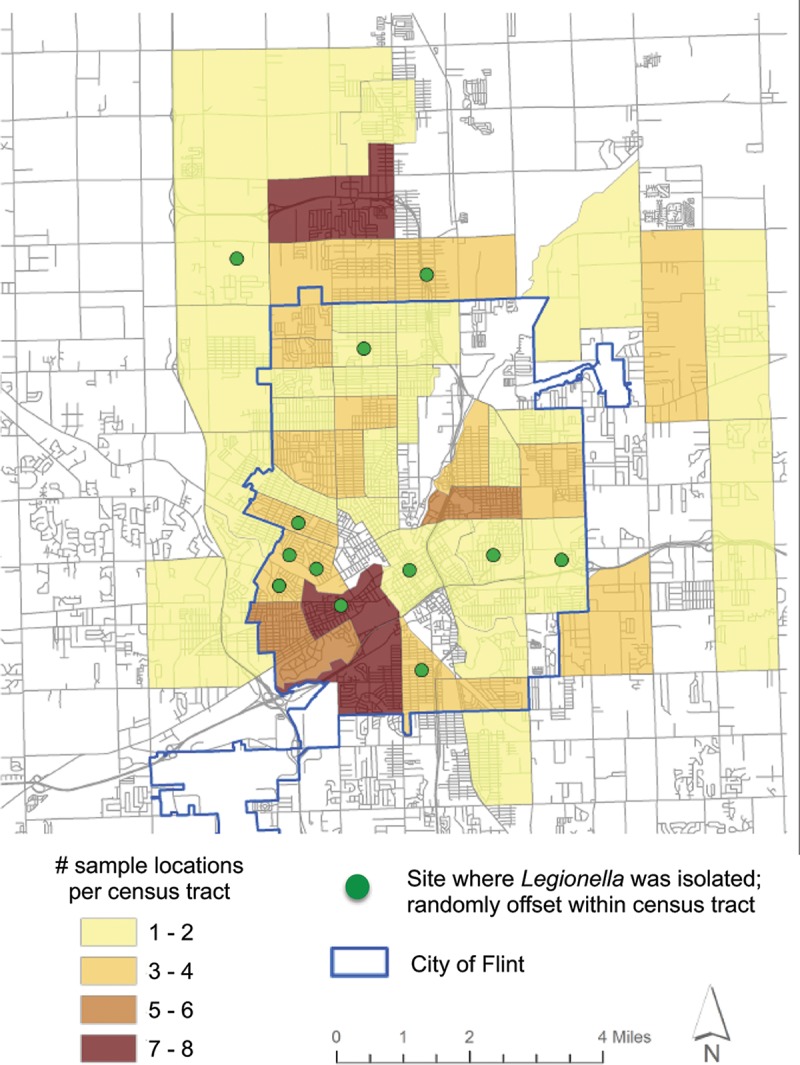
Geographic distribution of *Legionella* screening in Flint and neighboring census tracts. From 6 September through 29 October 2016, water samples were collected from the kitchen faucet, shower, and hot water heaters of 187 residences and processed to isolate viable *Legionella* spp. The number of sites sampled in each census tract is indicated (heat map). The sites where *Legionella* spp. were detected are indicated (green circles), with random offset within the census tract to protect residents’ privacy. The region serviced by the Flint municipal water supply is delineated (blue line).

To determine the prevalence of legionellae in Flint premise plumbing, a random sample of Flint’s households was surveyed from 6 September to 29 October 2016, 1 year after the 2015 summer outbreak. In Southeast Michigan, this period corresponds to the end of the typical Legionnaires’ disease season, which peaks annually during warm-weather months. From 130 residences, water samples were collected from the kitchen faucet, shower, and hot water heater and then cultured to isolate viable legionellae. Overall, *L. pneumophila* was cultured from 13 Flint and 2 Detroit residences (data not shown). Water samples from two other Flint residences contained non-*pneumophila* legionellae; these strains were not analyzed further. In one home, *L. pneumophila* was isolated from the hot water heater, shower, and kitchen faucet; in another residence, a positive culture was obtained from the shower and another from the kitchen faucet (Table 1). In total, we obtained a single *L. pneumophila* isolate from each of 16 positive premise plumbing samples from 13 Flint residences and a single *L. pneumophila* isolate from each of 2 premise plumbing sites in two Detroit residences (Fig. 1; Table 1). A similar incidence of culture-positive premise plumbing was obtained in Flint and non-Flint residences; however, the small sample size of non-Flint residences precludes statistical tests of significance. Of the hot water heaters that were sampled and noted, 125 were gas and only 1 was electric; therefore, our residential survey cannot address whether hot water heater design contributed to the risk of premise plumbing colonization by *L. pneumophila*, as reported previously (28).

**TABLE 1:** Southeast Michigan environmental and clinical isolates analyzed^a^

Strain	Source	Sero- group	Clonal complex	Sequence type	No. of isolates containing:							City	Site or references
					*flaA*	*pilE*	*asd*	*mip*	*mompS*	*proA*	*neuA*		
**EF1**	Environ	6	E	367	6	10	15	28	21	14	9	Flint	Kitchen faucet
EF2	Environ	6	E	367	6	10	15	28	21	14	9	Flint	Shower (A)
**EF3**	Environ	6	E	367	6	10	15	28	21	14	9	Flint	Hot water heater (A)
EF23	Environ	6	E	367	6	10	15	28	21	14	9	Flint	Kitchen faucet (A)
EF4	Environ	6	E	367	6	10	15	28	21	14	9	Flint	Kitchen faucet
EF5	Environ	6	E	367	6	10	15	28	21	14	9	Flint	Kitchen faucet
EF6	Environ	6	E	367	6	10	15	28	21	14	9	Flint	Kitchen faucet
EF7	Environ	6	E	367	6	10	15	28	21	14	9	Flint	Hot water heater
EF8	Environ	6	E	367	6	10	15	28	21	14	9	Flint	Hot water heater
**EF16**	Environ	6	E	367	6	10	15	28	21	14	9	Flint	Hot water heater
**ED20**	Environ	6	E	367	6	10	15	28	21	14	9	Detroit	Shower
EF22	Environ	6	E	367	6	10	15	28	21	14	9	Flint	Kitchen faucet
EF11	Environ	6	E	461	6	10	14	28	21	14	9	Flint	Kitchen faucet (B)
**EF13**	Environ	6	E	461	6	10	14	28	21	14	9	Flint	Shower (B)
**EF14**	Environ	6	E	461	6	10	14	28	21	14	9	Flint	Shower
EF15	Environ	6	E	461	6	10	14	28	21	14	9	Flint	Hot water heater
**ED21**	Environ	1		777	5	2	22	10	6	25	1	Detroit	Shower
**EF10**	Environ	1	D	1	1	4	3	1	1	1	1	Flint	Shower
**C45**	Clinic	1	D	1	1	4	3	1	1	1	1		
C51	Clinic	1	D	1	1	4	3	1	1	1	1		
C52	Clinic	1	D	1	1	4	3	1	1	1	1		
**C53**	Clinic	1	D	1	1	4	3	1	1	1	1		
C24	Clinic	1	A	222	2	19	5	10	18	1	10		
C25	Clinic	1	A	222	2	19	5	10	18	1	10		
C30	Clinic	1	A	222	2	19	5	10	18	1	10		
C32	Clinic	1	A	222	2	19	5	10	18	1	10		
C34	Clinic	1	A	222	2	19	5	10	18	1	10		
C35	Clinic	1	A	222	2	19	5	10	18	1	10		
C36	Clinic	1	A	222	2	19	5	10	18	1	10		
C38	Clinic	1	A	222	2	19	5	10	18	1	10		
C41	Clinic	1	A	222	2	19	5	10	18	1	10		
**C44**	Clinic	1	A	222	2	19	5	10	18	1	10		
**C29**	Clinic	1	A	213	2	19	5	10	18	1	2		
C31	Clinic	1	A	213	2	19	5	10	18	1	2		
C40	Clinic	1	A	213	2	19	5	10	18	1	2		
**C43**	Clinic	1	A	213	2	19	5	10	18	1	2		
C46	Clinic	1	A	213	2	19	5	10	18	1	2		
**C47**	Clinic	1	A	213	2	19	5	10	18	1	2		
C42	Clinic	1	A	213	2	19	5	10	18	1	2		
C49	Clinic	1	B	94	12	8	11	5	20	12	2		
C27	Clinic	1	B	1941	12	8	11	15	20	12	2		
C37	Clinic	1	B	1941	12	8	11	15	20	12	2		
C54	Clinic	1	B	1941	12	8	11	15	20	12	2		
C39	Clinic	1	B	44	4	8	11	10	10	12	2		
C26	Clinic	1	C	830	8	10	3	10	18	1	10		
C28	Clinic	1	C	1983	8	10	3	15	33	1	6		
C48	Clinic	1	C	62	8	10	3	15	18	1	6		
C56	Clinic	1	C	231	8	10	3	15	21	12	20		
C33	Clinic	1		45	5	1	22	26	6	10	12		
C50	Clinic	1		*10002*	12	14	16	1	15	13	2		
C55	Clinic	1		211	3	10	1	1	14	9	11		
Lp01	Lab	1		36	3	4	1	1	14	9	1		
Lp02	Lab	1		36	3	4	1	1	14	9	1		
ME4774	Environ	6	E	367								Murcia (Spain)	37, 38

a A boldface strain number indicates that it was selected for phenotypic analysis. Sites with the same parenthetical letter after them indicate that the samples came from the same residence. Environ, environment.

### Phylogenetic analysis of Southeast Michigan clinical and environmental *L. pneumophila* isolates.

To gain insight into the genetic diversity of the *L. pneumophila* strains obtained from premise plumbing in Flint in the fall of 2016, we determined the phylogenetic relationships among the 18 environmental isolates. In parallel, we also analyzed 33 clinical isolates that hospitals in three Southeast Michigan counties (Genesee, Wayne, and Oakland) submitted to the Michigan Department of Health and Human Services Bureau of Laboratories from 2013 to 2016 (Table 1). We first applied a DNA sequence-based typing (SBT) scheme that is standard practice in the international *Legionella* community (29). Using the DNA sequences of seven chromosomal alleles, SBT can discriminate among more than 2,000 different sequence types (STs) of *L. pneumophila* strains present worldwide, increasing the power of outbreak investigations (18). To identify related genotypes within the typed environmental and clinical *L. pneumophila* isolates from Southeast Michigan, we applied the eBURST algorithm (30). We defined a clonal complex as those strains that shared at least four of seven typing alleles with at least one other member of the group.

**FIG 2:**
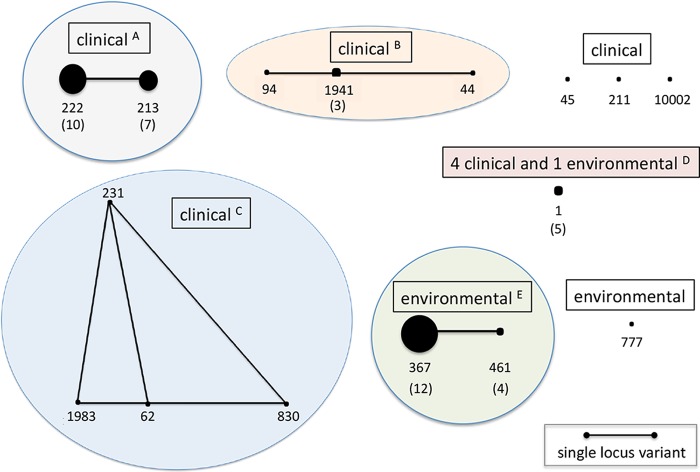
Phylogenetic relationship between Southeast Michigan clinical and environmental *L. pneumophila* isolates. The sequence types of 18 environmental isolates collected in the fall of 2016 and of 33 clinical isolates submitted to the MDHHS Bureau of Laboratories from Genesee, Wayne, and Oakland County hospitals in 2013 to 2016 were determined and their relationships identified by eBURST analysis. The number of isolates of each ST is indicated by the size of each filled black circle and is stated in parentheses if it is >1; the isolates’ origins (boxed) are also indicated. Clonal complexes (circled) were defined as members of an ST that shared at least four of the seven typing alleles with at least one other member of the group; superscripts refer to the complex designation in Table 1. The scale bar indicates the distance separating isolates that differ at one of the seven typing alleles.

Among the *L. pneumophila* strains isolated from patients in Southeast Michigan in 2013 to 2016, four phylogenetic clusters were evident, as judged by SBT and eBURST analysis. Of the 33 clinical isolates, 30 (91%) belonged to one of these four clonal complexes (Fig. 2). The largest group included 17 clinical isolates of the closely related ST222 and ST213, which differ at just one of the seven typing alleles. In the United States from 1982 to 2012, ST222 was among the most prevalent STs of both sporadic and outbreak-associated *L. pneumophila* SG1 isolates (18). The next-largest clinical cluster had five members of three related STs (ST1941, ST94, and ST44); a third cluster had four members, each of which was a singleton ST (ST231, ST1983, ST62, ST830). Four clinical isolates and one Flint environmental isolate were of ST1, the most common sporadic ST in the United States during the 1982–2012 period (18).

The majority of the *L. pneumophila* strains isolated from Flint premise plumbing in the fall of 2016 were closely related genetically. Of the 16 Flint strains, 15 (94%) formed a distinct clonal complex comprised of ST367 and ST461, which are identical at six of the seven typing loci. One of the Detroit premise plumbing isolates was also ST367; the other was a genetically distinct strain of ST777. Thus, compared to the 33 Southeast Michigan clinical isolates obtained during 2013 to 2016, the 18 *L. pneumophila* strains isolated from Flint and Detroit premise plumbing in the fall of 2016 were less genetically diverse, and only one environmental strain had an ST in common with the clinical strains.

### SG1 and SG6 *L. pneumophila* isolates verified by immunofluorescence microscopy.

**FIG 3:**
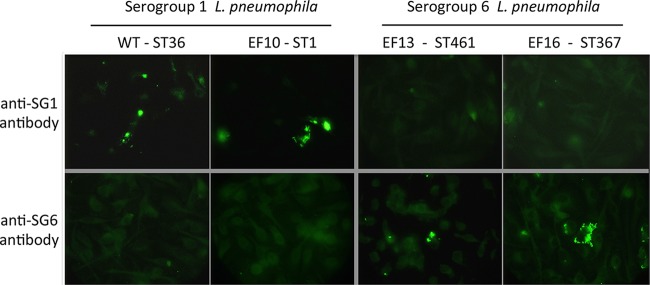
Serogroup 1 and serogroup 6 *L. pneumophila* isolates distinguished by immunofluorescence microscopy. Serogroup designations for clinical and environmental isolates, determined by clinical and commercial laboratories, respectively, were verified by immunolocalization of cell-associated bacteria 16 h after inoculation of mouse macrophage cultures using FITC-labeled serotype-specific antibodies. All bacteria and macrophages were visualized by staining their DNA with DAPI (not shown).

ST367 and -461 strains have been isolated previously from both patients and environmental surveillance operations during outbreaks in Poland, Israel, and Spain (31–33). Notably, these ST367 and -461 *L. pneumophila* strains were demonstrated to be of SG6. To determine whether the 16 Southeast Michigan premise plumbing *L. pneumophila* ST367 and -461 isolates were also of SG6, serotype-specific antibodies were used to perform immunocytochemistry (Pathogen Control Associates, Peachtree Corners, GA [data not shown]) and immunofluorescence microscopy. As expected, each of the ST367 and -461 *L. pneumophila* isolates from Flint and Detroit residences reacted with an SG6-specific antibody, whereas none cross-reacted with an antibody specific to SG1 antigens (Fig. 3 and data not shown).

### Some SG6 *L. pneumophila* strains are sensitive to human serum.

**FIG 4:**
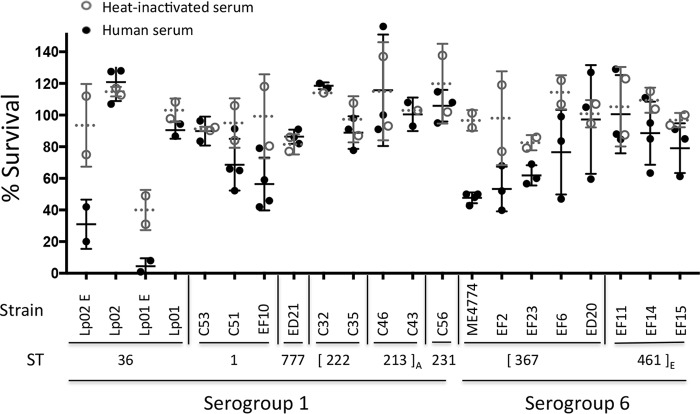
Sensitivities of serogroup 1 and serogroup 6 *L. pneumophila* isolates to human serum. The capacities of representative laboratory strains (Lp02, Lp01), clinical strains (“C” followed by a number), and environmental strains from Flint (“EF” followed by a number), Detroit (“ED” followed by a number), and Murcia, Spain (ME4774), to survive treatment with 90% human serum without (black circles) and with (gray circles) heat inactivation of complement were quantified by enumerating CFU before and after the 90- to 120-min incubation at 37°C. Shown are the means (solid or dotted horizontal lines) calculated in 2 to 4 independent experiments ± SD. Brackets indicate STs within clonal complex A or E (subscripts). For all strains, PE-phase cells were analyzed. Where indicated, exponential-phase cells (E) were also studied.

*L. pneumophila* serotyping is based on the structure of its lipopolysaccharide (LPS) O antigen, a feature of the outer membrane that also affects complement-mediated killing. Serogroup 1 *L. pneumophila* strains are resistant to killing by complement in human serum (34), whereas the serogroup 6 *L. pneumophila* Thunder Bay strain is sensitive, as judged by direct binding of the complement components C_3_ and C_9_, as well as decreased bacterial viability (35). To assess whether serogroup or strain origin affected the serum sensitivity of our Southeast Michigan strains, we incubated representative strains in nonimmune human serum or heat-inactivated human serum for 30 min at 37°C and then quantified viability by enumerating CFU. Consistently with the growth phase regulation of its LPS (36), the capacity of *L. pneumophila* to resist complement-mediated killing was growth phase dependent; serum killed >50% of exponential-phase cells of the serogroup 1 laboratory strains Lp01 and Lp02 but had little effect on the viability of cells cultured to the postexponential (PE) phase (Fig. 4). Complement-mediated killing accounted for most of the toxicity, since heat-treated serum had little effect on the viability of PE-phase bacteria. Although variability between strains and between experiments was observed, a few consistent patterns were noteworthy. Survival of ~90% or more was observed for PE-phase cells of six of the seven clinical serogroup 1 isolates (C53, C32, C35, C46, C43, and C56) and the serogroup 1 premise plumbing isolate from Detroit (ED21). In contrast, three of the five ST367 serogroup 6 strains were serum sensitive: two of these isolates were from Flint premise plumbing (EF2, EF23), and the third (ME4774) was isolated from a water facility during the 2001 outbreak in Murcia, Spain (37, 38). On the other hand, serum resistance was observed for two other ST367 strains (EF6, ED20) and three of the closely related ST461 serogroup 6 strains (EF11, EF14, EF15). Whether genetic differences within the genes encoding the LPS O antigen account for the degrees of serum sensitivity observed among and between the serogroup 1 and serogroup 6 strains analyzed here can be evaluated by genome sequencing.

### Both SG1 and SG6 *L. pneumophila* isolates infect macrophages efficiently.

According to an analysis of 3254 Legionnaires’ disease cases from 1980 to 1989 (39), mortality after infection with SG6 *L. pneumophila* exceeds that caused by SG1 strains (63% versus 37%, respectively). To cause disease, *L. pneumophila* survives and replicates within macrophages (40). Therefore, as a first step to gauge the virulence of the SG6 *L. pneumophila* strains isolated from Southeast Michigan premise plumbing, their capacity to survive ingestion by macrophages was quantified.

**FIG 5:**
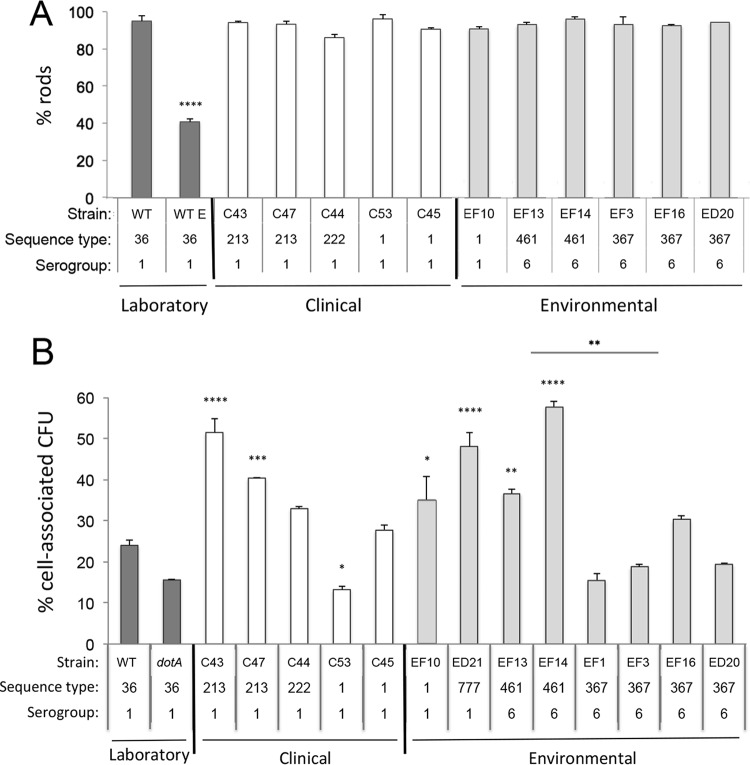
Survival of serogroup 1 and serogroup 6 *L. pneumophila* isolates 1 h after inoculation of macrophage cultures. (A) The capacity of representative clinical isolates (“C” followed by a number, white bars) and environmental isolates (light-gray bars) from Flint (“EF” followed by a number) and Detroit (“ED” followed by a number) to avoid degradation within mouse macrophages 1 h after infection with PE cells was determined by fluorescence microscopy using serotype-specific antibodies and PE- and exponential-phase cells (E) of strain Lp02 as positive and negative controls (dark-gray bars), respectively. Shown are means ± SD calculated from triplicate cell preparations from which at least 100 bacteria were scored as either rod or non-rod shaped. The data are representative of at least two independent experiments. (B) The capacity of each isolate indicated to bind to and survive on or within macrophages was determined for PE-phase cells by enumerating cell-associated CFU 1 h after inoculation of macrophage cultures. PE-phase wild-type Lp02 and *dotA* mutant cells served as positive and negative controls, respectively. Shown are means ± standard errors (SE) calculated from triplicate cell cultures. The origin, sequence type, and serogroup of each strain are indicated. One-way analysis of variance was calculated by comparing each strain to the WT laboratory strain: *, *P* < 0.05; **, *P* < 0.01; ***, *P* < 0.001; ****, *P* < 0.0001. The means for the two ST461 and four ST367 strains were compared using Welch’s t test (**, *P* < 0.01).

Macrophages derived from mouse bone marrow were infected for 1 h with representative clinical (C) and environmental (EF, from Flint; ED, from Detroit) isolates, fixed, and labeled with serotype-specific anti-*L. pneumophila* antibodies, and their DNA was stained. The infected macrophages were then examined by fluorescence microscopy. As expected, 95% of cell-associated PE-phase cells of *L. pneumophila* laboratory strain Lp02 remained as intact rods, whereas nearly 60% of the exponential-phase bacteria were degraded by macrophages (Fig. 5A) (41). As with the virulent PE-phase Lp02 control bacteria, for each of the clinical SG1 *L. pneumophila* strains, whether of ST1, ST213, or ST222, >85% of the PE-phase bacteria associated with macrophages remained rod shaped after a 1-h incubation. Likewise, >90% of the inocula of either SG1 or SG6 environmental strains of *L. pneumophila* evaded digestion by macrophages. The capacities to avoid degradation were similar for environmental strains of ST1, ST367, and ST461, as well as for isolates obtained from Flint and Detroit premise plumbing.

As a more rigorous test of infectivity, the capacity of representative clinical and environmental isolates to survive on or within macrophages was determined by enumerating cell-associated CFU 1 h after inoculation of the cultures. Four of the five clinical SG1 *L. pneumophila* strains survived their initial interaction with macrophages as well as or better than did the laboratory wild-type (WT) control strain (Fig. 5B). For example, 24% of the inoculum of the virulent PE-phase WT laboratory strain was cell associated and viable 1 h after addition to cultured macrophages, compared to 33 to 51% of the SG1 ST213 and ST222 clinical strains. Likewise, 1 h after infection, four of the environmental strains infected macrophages more efficiently than did the WT laboratory strain; 35 to 58% of the inoculum was viable and cell associated for the ST1 and ST777 SG1 strains and for both ST461 SG6 strains. Compared to ST461 strains, ST367 SG6 strains infected macrophages less efficiently (*P* < 0.01), with the infective cells ranging from 16 to 30% of each inoculum, a rate similar to that of the WT laboratory control strain. The single Detroit ST367 isolate (ED20) infected macrophages at a frequency similar to that of the three ST367 strains from Flint (EF1, EF3, and EF16). Thus, as judged by both direct and viable-cell-count assays, the clinical and environmental strains were competent to infect cultured mouse macrophages.

### SG1 and SG6 *L. pneumophila* isolates each replicate efficiently in macrophages.

**FIG 6:**
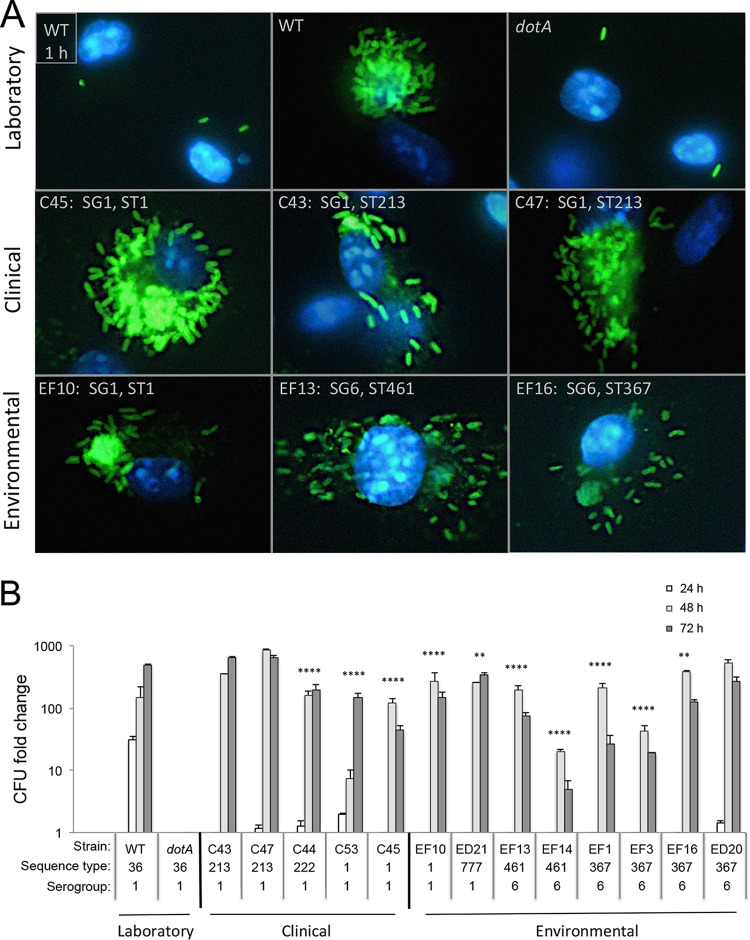
Yields of serogroup 1 clinical and serogroup 6 environmental isolates of *L. pneumophila* after infection of cultured macrophages. (A) Representative images of macrophages infected for 20 h with the indicated clinical isolates (“C” followed by a number) and environmental isolates from Flint (“EF” followed by a number) and Detroit (“ED” followed by a number) of the serogroup and sequence type shown and then fixed and stained with FITC-labeled and serotype-specific antibodies. PE-phase Lp01 *L. pneumophila* served as the positive control at both 1 and 20 h; the negative control was *dotA* mutant bacteria after 20 h. All bacteria and macrophages were visualized by staining their DNA with DAPI (not shown). Similar patterns were observed in two other independent experiments. (B) The capacity of representative clinical and environmental isolates from Flint and Detroit to replicate in mouse macrophages was quantified by enumerating total CFU per sample 1, 24, 48, and 72 h after infection. Shown are means calculated from duplicate or triplicate samples at each time indicated, divided by the mean cell-associated CFU 1 h after infection. Error bars indicate SD. One-way analysis of variance was calculated by comparing the maximum yield of each strain to that of the WT laboratory strain: **, *P* < 0.01; ****, *P* < 0.0001. Similar results were obtained in two additional independent experiments, with the exception of C53, which was analyzed twice.

The capacity of the environmental and clinical isolates to replicate within macrophages was first evaluated by microscopy. Infected macrophages were incubated for 20 h, fixed, and labeled with serotype-specific antibodies, and their DNA was stained. The WT laboratory strain replicated profusely during the primary infection period, unlike the negative-control strain, an avirulent *dotA* mutant (Fig. 6A). Likewise, numerous *L. pneumophila* progeny were visible within macrophages infected for 20 h with either of two different SG1 ST1 isolates, whether obtained from a patient (strain C45) or Flint premise plumbing (strain EF10). A similar pattern was observed for two SG1 ST213 clinical strains and representative SG6 ST461 and ST367 environmental strains.

To quantify *L. pneumophila* intracellular replication, CFU were enumerated 1, 24, 48, and 72 h after infection of mouse macrophages. During the 72-h infection period, the yield of each of the SG1 clinical strains, whether of ST213, ST222, or ST1, increased 120- to 850-fold (Fig. 6B). Some strain-to-strain variation was apparent; for example, the yield of the two ST1 isolates increased 150- and 120-fold, whereas that of the two ST213 isolates increased 650- and 850-fold. Similarly, the number of CFU increased by >100 for six of the eight environmental strains analyzed, a group that included strains of SG1 ST1 and -777 and SG6 ST461 and -367. A more modest ~50-fold increase in the number of CFU was observed for two strains (EF14 [SG6 ST461] and EF3 [SG6 ST367]). It was curious that seven of the eight environmental *L. pneumophila* strains achieved their maximal yield in CFU at 48 h, whereas two of the five clinical isolates and the laboratory strain did so at 72 h. Whether alterations to the bacteria and/or macrophages contribute to this kinetic difference requires more detailed analysis. Overall, regardless of their SG, ST, or origin in Flint or Detroit, each of the clinical and the environmental *L. pneumophila* isolates from Southeast Michigan had the capacity to infect, survive in, and replicate within cultured mouse macrophages, laboratory indicators of the potential to cause disease.

## DISCUSSION

Using a multidisciplinary approach coordinated with local partner health care providers, community organizations, and government agencies, the Flint Area Community Health and Environment Partnership (FACHEP) research team investigated the presence of *L. pneumophila* in Flint’s premise plumbing 1 year after Legionnaires’ disease outbreaks in two consecutive summers that coincided with the Flint water crisis. To do so, we integrated environmental monitoring and water testing with clinical education, assessment of social needs and resilience, population-based and molecular epidemiological surveillance, and laboratory assessments of virulence. In the molecular and phenotypic analysis of *L. pneumophila* strains isolated in Southeast Michigan reported here, we found little genetic overlap between the environmental and the clinical strains. All 33 clinical isolates are of SG1, consistent with the widespread use, both in Southeast Michigan and globally, of diagnostic tests that are specific to serogroup 1 *L. pneumophila*. In contrast, only two of the 18 premise plumbing isolates are SG1 strains. Instead, 89% of the 18 household strains are SG6 *L. pneumophila* of ST367 and ST416, two closely related STs. The virulence of the SG6 strains isolated from premise plumbing in Flint and Detroit resembles that of the SG1 clinical strains, as judged by their capacity to infect and replicate in cultured macrophages, a hallmark of *L. pneumophila* pathogenesis. As expected, SG6 *L. pneumophila* ST367 and -461 strains did not bind an SG1-specific antibody (Fig. 3). Since the widely applied diagnostic urinary antigen tests also rely on serotype-specific antibodies, it is likely that SG6 strains are not readily detected (42).

Across the globe, the most widespread *L. pneumophila* strains are SG1 ST1 strains. In China, SG1 ST1 strains represented 49% of the 164 *L. pneumophila* isolates collected from natural and engineered water supplies from 2005 to 2012 (43). Likewise, a U.S. survey analyzing 571 sporadic SG1 clinical isolates and 149 environmental isolates unrelated to disease cases that were collected from 1982 to 2012 found that ST1 *L. pneumophila* isolates were not only of the most prevalent serogroup (SG1) in both potable and nonpotable water (49% of all samples) but also the most frequent cause of sporadic Legionnaires’ disease (25% of cases) (18). Of the 4,785 clinical strains submitted from Europe to the EWGLI database, 10% are of ST1 (44). In France from 2008 to 2012, SG1 ST1 strains represented 21% of environmental isolates and 9.1% of clinical cultures yet accounted for 31% of nosocomial cases of Legionnaires’ disease (45). In a 2000–2008 surveillance study in England and Wales, *L. pneumophila* was cultured from 3.2% of samples collected from premise plumbing (46). Compared to 167 isolates from unrelated clinical cases, there was very little genetic overlap, as judged by ST (*P* < 0.0001). The only exception was SG1 ST1 strains, which comprised 20% of the premise plumbing isolates and 4.8% of the patient isolates (46). Likewise, in Southeast Michigan, SG1 ST1 was the only strain type isolated from both premise plumbing (1 of 18 samples; 5.6%) and patients (4 of 33 patients; 12%) (Fig. 2). Analysis of the whole-genome sequences of 229 *L. pneumophila* ST1 isolates from community- and hospital-acquired cases of Legionnaires’ disease and from water systems of the corresponding hospitals determined that ascribing an epidemiological link between ST1 strains requires a combination of molecular genetic and clinical epidemiological data, because levels of genetic diversity and stability differ across locations, and *L. pneumophila* spreads internationally (47). The molecular mechanisms and epidemiological processes that contribute to SG1 ST1 *L. pneumophila* prevalence worldwide remain to be understood.

Among the 33 SG1 clinical isolates collected in Southeast Michigan from 2013 to 2016, the majority belonged to one of four clonal complexes (Fig. 2). None of these clinical isolates were of ST35, ST36, or ST37, three strain types that caused a total of 10 outbreaks and 64 sporadic disease cases in the United States during 1982 to 2012 (18). In Southeast Michigan from 2013 to 2016, the largest clonal complex of clinical isolates included strains of ST222 and ST213, which are identical at six of the seven typing loci. In the United States from 1982 to 2012, *L. pneumophila* ST222 strains were among those most frequently associated with outbreaks, and strains of ST222 and ST213 were often associated with sporadic cases of Legionnaires’ disease (18). The ST222 genetic lineage has been prevalent in the northeastern regions of the United States and Canada but rare elsewhere (18, 48). After emerging in Ontario in 1999, over the next 8 years, ST222 *L. pneumophila* strains comprised 11 to 15% of clinical isolates in this Canadian province, including those responsible for the large 2005 outbreak at a long-term-care facility (48). The 2012 Calgary, Canada, outbreak of eight Legionnaires’ disease cases is an interesting exception, as it occurred during winter months with temperatures between 7°C and −26°C (49). Although environmental surveillance did not identify the reservoir, epidemiological and genome sequencing data predicted a likely point source: water contaminated with *L. pneumophila* of ST222 sprayed to extinguish fires or to minimize dust at construction sites in this city in western Canada (49).

It is possible that SG6 strains of *L. pneumophila* are endemic in the Great Lakes region. Of the 18 environmental isolates obtained in the fall of 2016 from Flint and Detroit premise plumbing, 89% were SG6 *L. pneumophila* strains. Among these, strains of the same ST, ST367, were isolated from 11 premise plumbing sites in Flint and one residence in Detroit, which did not receive treated Flint River water in 2014 to 2015. A 1981 survey of 52 sites in the Great Lakes, which border Michigan, yielded only two *L. pneumophila* strains, but both were of SG6 (50). In a 1984 analysis of water from 31 buildings in Ontario, a Canadian province adjacent to Southeast Michigan, SG6 strains were isolated slightly more frequently (12 of 19 buildings) than SG1 *L. pneumophila* strains (10 of 19 buildings). Elsewhere, SG6 strains are also prevalent. In the water distribution systems of nine hospitals in Poland, SG6 strains comprised 33% of the 11 environmental isolates, whereas SG1 strains accounted for 24% (31). In the Attica region of Greece, SG6 strains represented 62% of 37 *L. pneumophila* environmental isolates (51). In contrast, SG6 strains comprised only 15% of the *L. pneumophila* strains collected in England and Wales (46) and 11% of those isolated in France (52). More extensive surveillance studies can determine whether the predominance of SG6 *L. pneumophila* strains observed in Flint in the fall of 2016 is typical of the Great Lakes region or instead reflects a microbial response to the altered physiochemistry of the Flint municipal water system during 2014 and 2015. Another factor that might contribute to the predominance of SG6 strains in surveillance studies is *L. pneumophila*’s capacity to differentiate into a viable-but-nonculturable state. By this model, if, compared to other legionellae, SG6 strains are more readily cultured from water samples, their apparent prevalence would be elevated. Whether exposure to corrosive water characteristic of the 2014–2015 Flint water crisis alters the culturability, resilience, or virulence of *L. pneumophila* is one focus of ongoing analysis.

The genetic homogeneity of the 16 SG6 *L. pneumophila* strains cultured from Southeast Michigan premise plumbing is also striking: 12 strains are of ST367 and 4 others are of ST461, which share six of seven typing loci (Fig. 2). In contrast, of the 410 SG6 *L. pneumophila* strains submitted to the EWGLI database, only 5 are of ST367 and 9 are of ST461, and they originated from a variety of countries and continents. Whether the climate or water chemistry in Southeast Michigan contributed to the prevalence of this lineage during the fall of 2016 requires more detailed studies.

Although very few studies have reported sequence type analysis of SG6 *L. pneumophila* strains, the stability of particular genetic lineages has been suggested. In one hospital in Sweden, SG6 *L. pneumophila* isolates from three different patients and seven water samples were each of ST1392, as were eight of nine control clinical isolates from unrelated locations (53). In the Attica region of Greece, among 25 SG6 isolates, 28% were of ST68 and 20% were of ST461 (51), a type prevalent in Flint premise plumbing (Fig. 2) and also present in hospital water systems in Poland (31). In contrast, a 2-year surveillance study of a larger territory in Greece identified 10 different STs among 28 SG6 *L. pneumophila* strains isolated (51). More detailed genetic and phenotypic analyses of SG6 ST367 and ST461 strains from Flint, Detroit, Ontario, and Spain (33, 35) can evaluate whether this *L. pneumophila* lineage is either endemic to Southeast Michigan, more readily recovered from water samples, or more fit in the physiochemical conditions peculiar to the 2014–2015 Flint municipal water system.

A critical question is whether the SG6 *L. pneumophila* strains present in premise plumbing in Southeast Michigan put residents at risk. The virulence potential of environmental SG6 *L. pneumophila* ST367 and ST461 strains was comparable to that of SG1 clinical strains, as judged by quantifying entry, survival, evasion of digestion, and replication within cultured mouse macrophages (Fig. 5 and 6). In Israel, Spain, and Greece, SG6 strains of ST367 and ST461 have been isolated from patients as well as from corresponding environmental surveillance efforts (32, 33, 51). Moreover, in a phylogenetic analysis of 73 clinical non-SG1 *L. pneumophila* isolates in Ontario, which borders Southeast Michigan, SG6 was most prevalent (47%) (35). In a 1990 study in Sweden, SG6 was also the most common non-SG1 *L. pneumophila* serogroup isolated from Legionnaires’ disease patients (54). In the United States, a 1980–1989 survey of 327 Legionnaires’ disease patients attributed 6.4% of disease cases to SG6 *L. pneumophila*, second only to SG1 (62%). However, the 64% mortality rate of patients with SG6 infection exceeded that for patients infected with SG1 *L. pneumophila* (37%), even after controlling for specific underlying conditions, age, nosocomial acquisition, and other risk factors (39). On the other hand, a 2013 laboratory analysis discovered that the SG6 *L. pneumophila* Thunder Bay strain replicates more efficiently in *Acanthamoeba castellanii* and human monocyte-like cells than SG1 strains yet is more sensitive to killing by the innate immune system’s alternative complement pathway (35). In a mouse model of infection, these SG1 and SG6 strains replicate to similar numbers in the lung, but the SG6 Thunder Bay strain was less proficient at disseminating into the blood and other tissues (35). Accordingly, infection and replication in cultured mouse macrophages may not accurately predict virulence in human lung.

Other key unanswered questions concern *L. pneumophila* fitness in water and aerosols, the major route of human infection. It is not known whether survival in water aerosols by SG6 *L. pneumophila* is comparable to that of SG1 strains, whose unusual LPS includes a lipid A moiety that is especially hydrophobic (55). Since the LPS of SG1 *L. pneumophila* also contributes to lysosome evasion (36), SG1 and -6 strains may differ in their capacities to survive within phagocytic protozoa, their natural predators (56).

Because the widely applied urinary antigen test is specific for SG1 *L. pneumophila* (42), Legionnaires’ disease due to SG6 and other non-SG1 strains is more difficult to diagnose. Compared to the 60 to 80% sensitivity of the urinary antigen test, the sensitivity of respiratory specimen culture methods ranges from 20 to 80% (57). Furthermore, obtaining sputum specimens from pneumonia patients is challenging, and diagnosis by culture takes several days, requires specialized media and trained personnel, and is hindered by prior empirical treatment with antibiotics (7, 57). Consequently, <5% to 12% of Legionnaires’ disease cases in Europe and the United States are confirmed by culture (4, 5, 9). Instead, urinary antigen testing has been enlisted to confirm diagnosis of >90% of Legionnaires’ disease cases in both the United States and Europe (7, 9). Indeed, although pneumonia due to non-SG1 *L. pneumophila* likely accounts for >10% of total Legionnaires’ disease incidence (51), it is generally understood that non-SG1 cases are underdiagnosed and underreported (3, 7, 9, 10). In Flint’s Genesee County in 2014 to 2015, the average number of deaths due to pneumonia increased 43% above the average of the previous 5 years; in contrast, pneumonia deaths across Michigan increased only 6% above the state’s 5-year average (58). During this period in Southeast Michigan, it was extremely rare that a Legionnaires’ disease case that was negative by the urinary antigen test was also analyzed by either culture or direct fluorescence antibody testing, and none were analyzed by serology or PCR. Accordingly, during the 2014–2015 Genesee County outbreak, there is no clinical microbiology laboratory evidence of Legionnaires’ disease due to non-serogroup 1 *L. pneumophila*. Whether SG6 *L. pneumophila* strains present in Flint premise plumbing contributed to the 2014–2015 increase in pneumonia in Genesee County could be investigated by an epidemiological study that incorporates serological testing for SG6-specific antibodies in patients with community-acquired pneumonia of unknown etiology. Until rapid, sensitive tests are developed to identify and type non-SG1 strains of *L. pneumophila*, clinicians and public health professionals must rely on culture-based methods to identify cases or sources of Legionnaires’ disease, a critical step in protecting the public from this illness, the most common associated with drinking water in the United States.

## MATERIALS AND METHODS

### Environmental surveillance.

To evaluate the prevalence of legionellae in Flint premise plumbing, of the 187 total residences enrolled in the study, 130 were sampled and their water cultured according to the protocol described here. The majority (127) of homes were selected at random from Genesee County from tax parcel records; three other residences were selected from a list of potential self-identified participants, with preference given to residents based on perceived risk of acquiring Legionnaires’ disease. Once residences were selected, institutional review board (IRB)-approved recruitment letters were mailed to each property. Within 2 weeks, study personnel knocked on residents’ doors and attempted to obtain informed consent. If consent was obtained, a three-person team returned to collect environmental samples, complete an epidemiological survey focused on legionellosis risk factors, perform a psychosocial needs and resilience assessment, and provide referrals to social services if necessary.

From these 130 residences, a total of 357 water and biofilm samples were collected. At each location, when possible, 2-liter water samples were collected from the hot water tank drain valve, the primary shower used by residents, and the kitchen sink after 5 min of flushing (bypassing point-of-use filters when present). Each 2-liter water sample was collected into polyethylene terephthalate glycol (PETG) bottles containing 0.5 ml of 0.1 N sodium thiosulfate to remove any residual chlorine. After gentle mixing, 125 ml of the aqueous sample was placed in a sterile high-density polyethylene (HDPE) bottle and shipped overnight to Pathogen Control Associates (Peachtree Corners, GA) for culturing. The remaining portion of the 2-liter sample was delivered to Henry Ford Hospital for further analysis (data not shown). To collect shower samples, the shower head was removed prior to the 2-liter first-draw water sample being collected, using hot water only. Once the shower water was collected, a biofilm sample was collected from the shower pipe neck by using a sterile 6-inch swab with a polyester tip, which was then placed in a 15-ml sterile Falcon tube with 5 ml of phosphate-buffered saline (PBS).

### *Legionella* isolation from water samples.

Water samples collected from the residential surveillance operation were cultured for legionellae by Pathogen Control Associates personnel using methods compatible with the relevant CDC laboratory protocol (59) and the International Organization for Standardization 11731:1998 standard (60). To identify suspect colonies to the genus level, colony characteristics were examined microscopically, and their requirement for l-cysteine was tested. To identify candidate isolates to the species and serogroup levels, monovalent and polyvalent direct fluorescent-antibody reagents were used in the direct fluorescence antibody and/or slide agglutination test as described previously (61, 62). To eliminate the risk of experimental bias, premise plumbing samples were cultured by staff of Pathogen Control Associates Laboratories, who then transferred all legionella isolates to the Henry Ford Hospital System for coding, storage, and transfer to the University of Michigan for analysis (Table 1). From each culture-positive premise plumbing sample, only one *L. pneumophila* isolate was obtained and characterized. Therefore, one limitation of our study is that it cannot address how often residences are colonized by more than one strain of *L. pneumophila*, as recommended by ISO 11731:1998 (60).

### Clinical isolates.

Via a material transfer agreement, the Michigan Department of Health and Human Services Bureau of Laboratories provided 33 clinical isolates submitted from 2013 to 2016 by hospitals in Genesee, Wayne, and Oakland Counties in Michigan (Table 1). All specimens were deidentified prior to shipment to the University of Michigan Medical School.

### Laboratory control strains, culture conditions, and reagents.

*L. pneumophila* strain Philadelphia-1 derivative Lp02, a thymidine auxotroph, served as a previously characterized laboratory reference strain. All *L. pneumophila* strains were cultured at 37°C in *N*-(2-acetamido)-2-aminoethanesulfonic acid (ACES; Sigma)-buffered yeast extract broth (AYE); strain Lp02 cultures were supplemented with 100 μg/ml thymidine (T; Sigma). To quantify CFU, aliquots were plated on ACES-buffered charcoal-yeast extract agar (CYE) (63) and incubated at 37°C for 4 days; medium for strain Lp02 was supplemented with 100 μg/ml thymidine. Bacteria obtained from CYE (T) were cultured overnight in AYE (T) and then diluted and cultured overnight to obtain cells in exponential (optical density at 600 nm [OD_600_] of 1.2 to 1.8) or postexponential (PE) (OD_600_ of 3.2 to 3.9) phase (64).

### Sequence-based typing.

Genotypes were assigned using the sequence-based typing protocol developed by the European Working Group for *Legionella* Infections (65, 66). Briefly, after extracting genomic DNA from an isolated colony, seven designated gene segments (*flaA*, *pilE*, *asd*, *mip*, *mompS*, *proA*, and *neuA*) were amplified by PCR. After purification, the DNA products were sequenced using the designated forward and reverse primers (65, 66). To assign allele numbers, each DNA sequence was trimmed *in silico* by the *Legionella* SBT quality tool (http://www.hpa-bioinformatics.org.uk/cgi-bin/legionella/sbt/seq_assemble_legionella1.cgi) and then used to query the *L. pneumophila* database (http://www.hpa-bioinformatics.org.uk/legionella/legionella_sbt/php/sbt_homepage.php). Together, the list of seven assigned allele numbers defines each strain’s numerical sequence type (e.g., ST1). Novel alleles and STs were submitted to the ESGLI database. SBT analysis was performed on deidentified samples at the University of Michigan. Likewise, 33 clinical isolates were deidentified, coded, and shipped by the MDHHS to the University of Michigan for blind SBT analysis.

### Serotyping.

The serogroup designations for clinical and environmental isolates initially determined by clinical and commercial laboratories, respectively, were verified by immunolocalization of cell-associated bacteria 1 h after inoculation of mouse macrophage cultures using fluorescein isothiocyanate (FITC)-labeled serotype-specific antibodies. An *L. pneumophila* SG1-specific rabbit polyclonal antibody (Pierce PA1-73140) was diluted 1:1,000, and an *L. pneumophila* SG6-specific antibody (prepared by the CDC and a gift of Paul Edelstein of the University of Pennsylvania School of Medicine) was diluted 1:200. All bacteria and macrophages were visualized by staining their DNA with DAPI (4′6-diamidino-2-phenylindole) (not shown).

### Phylogenetic analysis.

Phylogenetic relationships among the 18 environmental and 33 clinical *L. pneumophila* isolates were evaluated using the v3 eBURST online tool (http://eburst.mlst.net/). We defined a clonal complex as strains of those STs that share at least four of the seven alleles with at least one other member of the group.

### Immunofluorescence microscopy.

Microscopy was performed by culturing 2.5 × 10^5^ macrophages on 12-mm glass coverslips overnight prior to infection with PE-phase bacteria at a multiplicity of infection of 1. After the infection period, cultures were fixed and stained as described previously (41) using an *L. pneumophila* SG1-specific rabbit polyclonal antibody conjugated to FITC (Pierce; PA1-73140), which was diluted 1:1,000, and the FITC-conjugated *L. pneumophila* SG6-specific antibody (prepared by the CDC and a gift of Paul Edelstein), which was diluted 1:200. All bacteria and macrophages were identified by labeling them with the DNA stain DAPI, which was purchased as ProLong Gold antifade reagent (Molecular Probes) and included in the mounting medium.

### Serum sensitivity.

To quantify the sensitivity of *L. pneumophila* to human complement, human serum was purchased (Fisher Scientific; BP2657, lot 174248) and stored at −20°C. As a negative control, complement was heat inactivated by incubating an aliquot of serum at 56°C for 30 min just before use. PE-phase *L. pneumophila* cells (OD_600_, 3.5 to 4.1) were collected by centrifugation of 600-μl culture aliquots at 5,000 rpm for 5 min, washed once with 1 ml of PBS, and resuspended in 0.5 ml of PBS. After dilution of duplicate 75-μl cell aliquots into 5 ml of PBS, 50 μl of cells was diluted into 450 μl of nonimmune or heat-inactivated human serum to achieve a final concentration of approximately 5 × 10^6^ cells per ml of 90% serum. After a static incubation at 37°C for 90 to 120 min, cells were serially diluted in PBS and then plated in duplicate on CYE to enumerate CFU. In parallel, the CFU of each untreated inoculum was enumerated after serial dilution of duplicate aliquots. For each strain, the percent survival was calculated as (CFU in serum/CFU in inoculum) × 100 from the mean of results from at least six aliquots per experiment in 2 to 4 independent experiments.

### Intracellular growth.

The growth of *L. pneumophila* strains in bone marrow-derived macrophages from A/J mice (Jackson Laboratories) was assessed by enumerating CFU on CYE (T) medium essentially as described previously (67). PE-phase wild-type Lp01 and *dotA* mutant cells served as positive and negative controls, respectively. Shown are means ± standard deviations (SD) calculated from triplicate cell preparations. The sequence type, serogroup, and origin of each strain are indicated in Table 1.
